# Supplementary material for: Normative serum cortisol levels in second and third trimesters and their associated factors: A prospective cohort study from Sri Lanka
Source: PLoS One. 2025 Sep 8;20(9):e0322685. doi: 10.1371/journal.pone.0322685 (PMC12416640; doi:10.1371/journal.pone.0322685)
Supplement: Attachment — Submitted filename: Response letter.docx [file pone.0322685.s002.docx]

**Response letter**

The authors are highly appreciative and grateful for the excellent review process. We are delighted with the constructive, encouraging, and insightful comments of the editor and the reviewers. We strongly believe that the comments of the editor and the reviewers greatly enhanced the quality and the scientific impact of our manuscript. Furthermore, we are thankful for the comments to improve the comprehensibility and the understandability of our manuscript. We addressed all the comments and enhanced our manuscript as suggested.

| **Comment** | **Response** | **Page and Line** |
| --- | --- | --- |
| However, there is some ambiguity regarding the timing of participant recruitment. While it is stated under the "Procedure" section (line 153) that recruitment occurred during the first trimester, it is also mentioned (lines 146–147) that serum cortisol levels were available only for 1,290 participants who attended follow-up visits in the second or third trimester. Although the authors refer to a previously published methodology, it would greatly improve the clarity of the current manuscript to explicitly state when participants included in this analysis were initially recruited.  Although the authors refer to a previously published methodology, it would greatly improve the clarity of the current manuscript to explicitly state when participants included in this analysis were initially recruited. Clear delineation of the recruitment timeline would aid in understanding the study design and cohort selection. | The authors highly appreciate the insightful and constructive comments of the academic editor. We edited the methodology section as suggested by the editor.  “Study population  The target population comprised pregnant women residing in the Anuradhapura district, North Central Province, Sri Lanka, who were eligible for enrolment based on the following criteria: (i) registration with the national maternal care program between 1 July and 30 September 2019, (ii) confirmed pregnancy with a period of amenorrhea less than 12 weeks at the time of registration, and (iii) permanent residence within the study district. The national maternal care program achieves over 95% population coverage, enabling near-complete enrolment of eligible pregnancies into antenatal care services. [28].”  “Study sample  A total of 3,374 pregnant women were recruited for the original cohort study [29]. Of these, 1,450 participants attended the first follow-up visit, which occurred during the late second or early third trimester. Among those who attended the follow-up, 1,290 (89.0%) underwent serum cortisol testing. At this follow-up, gestational age was re-estimated based on first-trimester dating ultrasound scans. For the present analysis, a subset of 1,010 women with recalculated gestational ages between 25 and 29 weeks at the time of cortisol sampling was selected.” | Page  Line 122-137 |
| I read the presented manuscript with great interest. I wish to commend the authors in presenting a large data set from a LMIC set up which is a difficult task to accomplish. The study presents descriptive data of maternal serum cortisol levels in a large cohort of pregnant women in the second trimester. I have only a few comments to make. 1. Please highlight the fact that ALL women in the cohort had their cortisol levels with-in the accepted reference range for the gestation. | The authors are grateful for the critical and encouraging comments to improve the quality of our manuscript. We edited the conclusion of the manuscript to explicitly state that the serum cortisol level did not exceed the upper limit, in addition to stating it in the abstract, results, and the discussion.  **Conclusion**:  “The mean serum cortisol level in all pregnant women was 10.93(±3.83) µg/dL, with none of the participants exceeding the upper limit and with no significant difference between singleton and twin pregnancies.”  **Abstract**:  “None of the study participants had a cortisol level exceeding the upper limit of 42 µg/dL, and 464 (45.9%) had levels less than 10 µg/dL.”  **Results**:  “None of the study participants had a cortisol level exceeding the upper limit of 42 µg/dL, and 464 (45.9%) had levels less than 10 µg/dL, with 236 (50.9%) in the second trimester and 228 (49.1%) in the third trimester.”  **Discussion**:  “The mean serum cortisol level in the study sample was close to the lower limit, with none of the study participants exceeding the upper limit of the standard levels [32].” | Page 22  Lines 355-357  Page 3  Lines 59-61  Page 12  Lines 201-203  Page 18  Lines 272-274 |
| I agree with the authors that the relationship with EPDS score and maternal serum cortisol levels may not have shown a correlation due to the chronology of the measurements. Please make a clear statement of when the EPDS score was obtained and when the serum cortisol level was measured- what was the time difference between the two measurements? | Authors are thankful for the insightful comments of the reviewers to improve the quality and the understandability of our manuscript. As suggested, we included when the EPDS test was administered to enable the understanding of the time difference between the two measurements.  **Methodology:**  “During the follow-up visit between 25 to 29 weeks of POG, fasting early morning serum cortisol levels were obtained …”  “The validated and culturally-adopted Edinburgh Postnatal Depression Scale (EPDS) was used to assess antenatal depression in the first (POG less than 13 weeks) and second visits (POG 25 to 29).” | Page 8  Lines 146-148  Page 8  Lines 150-152 |
| If the authors can present pregnancy outcome data, please check for fetal outcomes in relationship with second trimester cortisol levels - eg, previous authors have presented a relationship with high normal (>17) second trimester cortisol levels with adverse fetal outcomes. (The data will be an useful addition for a systematic review/ meta analysis) | The authors are highly appreciative of this constructive suggestion to further improve the value of our manuscript. We included a subsection on pregnancy outcomes and the serum cortisol levels.  “Pregnancy outcomes  Pregnancy outcomes were available for 990 participants (98.0%), including 985 live births (99.5%), 3 stillbirths (0.3%), and 2 intrauterine deaths ≥28 weeks of gestation (0.2%). Among live births, 4 early neonatal deaths (<7 days) and 5 infant deaths (<12 months) were reported. Preterm birth occurred in 208 cases (21.1%), including 10 very preterm (1.0%) and 3 extreme preterm deliveries (0.3%). Mean serum cortisol levels did not differ significantly between preterm (11.14 ± 3.74 µg/dL) and term births (10.87 ± 3.87 µg/dL; p = 0.372). Of 968 births with available weight data, 153 (15.8%) were low birth weight, 4 (0.4%) were very low birth weight, and none were extremely low birth weight. Cortisol levels were comparable between low birth weight (10.87 ± 3.53 µg/dL) and normal birth weight deliveries (10.94 ± 3.91 µg/dL; p = 0.837).” | Page 18  Lines 258-268 |
| Thank you for conducting this research and comprehensive article. This gives eye open for pregnancy and its related factors in developing country. I thought increase in cortisol levels in psychological stresses, but it was not. Very interesting article. Best wishes for future endeavours | The authors are glad and appreciative of the encouraging, constructive, and supportive comments of the reviewer. |  |
